# Integrated data analysis reveals potential drivers and pathways disrupted by DNA methylation in papillary thyroid carcinomas

**DOI:** 10.1186/s13148-017-0346-2

**Published:** 2017-05-02

**Authors:** Caroline Moraes Beltrami, Mariana Bisarro dos Reis, Mateus Camargo Barros-Filho, Fabio Albuquerque Marchi, Hellen Kuasne, Clóvis Antônio Lopes Pinto, Srikant Ambatipudi, Zdenko Herceg, Luiz Paulo Kowalski, Silvia Regina Rogatto

**Affiliations:** 1 International Research Center-CIPE–A.C. Camargo Cancer Center and National Institute of Science and Technology in Oncogenomics (INCiTO), São Paulo, Brazil; 2 Department of Urology, Faculty of Medicine, UNESP, Sao Paulo State University, Botucatu, São Paulo Brazil; 3 Department of Pathology, A.C. Camargo Cancer Center, São Paulo, SP Brazil; 4 Epigenetics Group; International Agency for Research on Cancer (IARC), Lyon, France; 5 Department of Head and Neck Surgery and Otorhinolaryngology, A. C. Camargo Cancer Center, São Paulo, SP Brazil; 6 Department of Clinical Genetics, Vejle Hospital and Institute of Regional Health Research, University of Southern Denmark, Kabbeltoft 25, Vejle, 7100 Denmark

**Keywords:** Papillary thyroid cancer, DNA methylation, Integrative analysis, FGF signaling pathway, Retinoic acid pathway, *BRAF*V600E mutation

## Abstract

**Background:**

Papillary thyroid carcinoma (PTC) is a common endocrine neoplasm with a recent increase in incidence in many countries. Although PTC has been explored by gene expression and DNA methylation studies, the regulatory mechanisms of the methylation on the gene expression was poorly clarified. In this study, DNA methylation profile (Illumina HumanMethylation 450K) of 41 PTC paired with non-neoplastic adjacent tissues (NT) was carried out to identify and contribute to the elucidation of the role of novel genic and intergenic regions beyond those described in the promoter and CpG islands (CGI). An integrative and cross-validation analysis were performed aiming to identify molecular drivers and pathways that are PTC-related.

**Results:**

The comparisons between PTC and NT revealed 4995 methylated probes (88% hypomethylated in PTC) and 1446 differentially expressed transcripts cross-validated by the The Cancer Genome Atlas data. The majority of these probes was found in non-promoters regions, distant from CGI and enriched by enhancers. The integrative analysis between gene expression and DNA methylation revealed 185 and 38 genes (mainly in the promoter and body regions, respectively) with negative and positive correlation, respectively. Genes showing negative correlation underlined FGF and retinoic acid signaling as critical canonical pathways disrupted by DNA methylation in PTC. *BRAF* mutation was detected in 68% (28 of 41) of the tumors, which presented a higher level of demethylation (95% hypomethylated probes) compared with *BRAF* wild-type tumors. A similar integrative analysis uncovered 40 of 254 differentially expressed genes, which are potentially regulated by DNA methylation in *BRAF*V600E-positive tumors. The methylation and expression pattern of six selected genes (*ERBB3*, *FGF1*, *FGFR2*, *GABRB2*, *HMGA2*, and *RDH5*) were confirmed as altered by pyrosequencing and RT-qPCR.

**Conclusions:**

DNA methylation loss in non-promoter, poor CGI and enhancer-enriched regions was a significant event in PTC, especially in tumors harboring *BRAF*V600E. In addition to the promoter region, gene body and 3’UTR methylation have also the potential to influence the gene expression levels (both, repressing and inducing). The integrative analysis revealed genes potentially regulated by DNA methylation pointing out potential drivers and biomarkers related to PTC development.

**Electronic supplementary material:**

The online version of this article (doi:10.1186/s13148-017-0346-2) contains supplementary material, which is available to authorized users.

## Background

Thyroid cancer is the most common tumor of the head and neck region, with the highest incidence among the endocrine neoplasias [1]. Papillary thyroid cancer (PTC) is the histological subtype with higher incidence (80% of cases) worldwide [2].

The thyroid carcinogenesis involves a constitutive activation of two major pathways associated to tyrosine-kinase, including mitogen-activated protein kinase (MAPK) and phosphatidylinositol 3-kinase (PI3K) [3]. The activation of these pathways occurs mainly due to point mutations in *BRAF* and *RAS* and chromosomal rearrangements in *RET* [3]. MAPK signaling pathway activated by genetic alterations is frequently involved in cell proliferation, growth, and survival [4].

Approximately 60% of PTC cases are characterized by T1799A *BRAF* transversion nucleotide change (over 95% of the mutations), resulting in V600E mutant protein with constitutive activation of BRAF kinase [5–7]. *BRAF* mutation has been associated with unfavorable prognosis including large primary tumors, lymph node and vascular invasion, advanced stage, extrathyroidal extension, distant metastases, and recurrence [8, 9]. However, there are no consensus in literature, since many studies have not found this association [10–12].

The methylation pattern of several genes has been assessed in PTC, and most of them plays a role in thyroid gland function (*TSHR*) [13] and iodine metabolism (*NIS* and *SLC26A4*) [14] or acts as a tumor suppressor gene (*RASSF1A*, *TIMP3*, and *RARβ2*) [15, 16]. In addition, an association between *BRAF*V600E mutation and aberrant DNA methylation profile has been reported in thyroid cancer [17–21].

Recently, large-scale approaches were used to investigate the methylation profile of thyroid cancer [17–23]. These technologies allow the assessment of not only CpG islands (CGI) and promoter regions, as previously reported, but unveiled novel regions involved in neoplastic process, such as CGI shores/shelf, non-CGI promoter and enhancers [24, 25]. However, the methylation as a regulatory mechanism of gene expression is poorly explored in PTC, even in the multiplatform robust study performed by The Cancer Genome Atlas (TCGA) [21].

To our knowledge, the current study is the first to assess a substantial matched-sample subset with methylation and expression data addressing the available data from TCGA study to cross-validate the results. The genes signature potentially regulated by methylation inferred the role of this epigenetic event in PTC development.

## Methods

### Sample population

Snap frozen PTC samples stored at tissue biobank of the A.C. Camargo Cancer Center, SP, Brazil, were obtained retrospectively. Forty one papillary thyroid carcinomas of patients treated with total thyroidectomy followed by radioiodine therapy and matched non-neoplastic adjacent tissues (NT) samples were included in this study. Cases with incomplete clinical data in medical records or diagnosed with previous or synchronic malignancies were excluded (except basal skin cell carcinoma). Clinical and histopathological data are summarized in Table 1.

**Table 1:** Clinicopathological features of 41 patients diagnosed with papillary thyroid carcinoma

Characteristics	Number	Frequency (%)
Age (years)		
Median (interquartile range)	39(20-77)	
<55	34	83
≥55	7	17
Gender		
Female	30	73
Male	11	27
Size Tumor (cm)		
Median (range)	1.2(0.6-3.2)	
*mPTC* (≤1)	18	44
PTC (>1)	23	56
Predominant variant		
Classic	29	71
Follicular	7	17
Other^a^	3	7
Not available	2	5
Multicentricity		
No	17	41
Yes	24	59
Extrathyroidal extension		
No	20	49
Yes	21	51
Lymph Nodes involvement		
No	25	61
Yes	16	39
Angiolymphatic invasion		
No	39	95
Yes	1	5
Perineural invasion		
No	36	88
Yes	2	5
Not available	3	7
Outcome		
Favorable^b^	36	88
Poor ^c^	5	12
Follow-up		
>5 years	36	88
<5 years	5	12
Somatic alterations		
* BRAF* mutation	28	68
* BRAF* wild-type	13	32
* RAS* mutation	0	0
* RAS* wild-type	41	100
RET/PTC inversion	5	12
* RET*/PTC wild-type	36	88

*mPTC* Papillary thyroid microcarcinoma

^a^Three rare variants were grouped: one tall cells, one oncocytic, and one mucosecretory

^b^Patients without any suspicion of active disease by imaging scan and/or serum thyroglobulin measurement in at least 5 years of follow-up

^c^Patients with confirmed recurrent disease in the follow-up

### Nucleic acids extraction and analysis of somatic mutations

Genomic DNA extraction was performed according to conventional protocol using enzymatic degradation with proteinase K followed by purification with organic solvents (phenol/chloroform). RNA was isolated as previously reported [26].

Somatic point mutations of *BRAF* (codon 599), *KRAS* (codon 12/13), *HRAS* (codon 61), and *NRAS* (codon 61) were evaluated by pyrosequencing using a Pyromark Q96 ID system (Qiagen, Valencia, CA, USA). *RET* rearrangements (*RET*/PTC1 and *RET*/PTC3) were detected by RT-qPCR on a 7500 Real Time PCR System (Applied Biosystems, Foster, CA, USA) (detailed in Additional file 1).

### DNA methylation and gene expression profiling

Five hundred nanograms of DNA (*Qubit® dsDNA BR Assay no Qubit® 2.0* Fluorometer (Life Technologies, Carlsbad, CA, USA) were bisulfite-modified using EZ-DNA Methylation-Gold Kit (Zymo Research, Irvine, CA, USA) according to the manufacturer’s recommendations. Converted DNA was used for the genome-wide methylation assays (Infinium Human Methylation450 BeadChip array-Illumina, San Diego, CA, USA). Arrays were scanned by HiScan system (Illumina), and methylation data were analyzed as β values. Genome-wide DNA methylation data processing was done as reported previously [27] (detailed in Additional file 1). Limma package [28] was used to identify significant probes adopting adjusted (Bonferroni) *p* value <0.05 and mean delta β value (Δβ) of 0.15 as a threshold for differential DNA methylation. A hypergeometric test (*p* < 0.05) was performed from *phyper* function of *STATS* package in *R* language to compare differentially methylated probes in relation to genomic regions (Illumina 450K array annotation).

Gene expression data were obtained from our previously reported study (GEO accession number GSE50901) [26]. The unsupervised hierarchical clustering analysis was performed using the most variable probes (interquartile range >0.2 to methylation and >1.0 to gene expression). Euclidean distance with average linkage method was used in all clustering analysis by BRB array tool software (https://brb.nci.nih.gov/BRB-ArrayTools/download.html). Student *t* test was assessed to verify the association between methylation/expression data of selected genes with clinical parameters.

### Integrative analysis and cross-study validation

All probes differentially methylated (|Δβ| 0.15 and adjusted *p* < 0.05) and expressed (FDR <5% and fold change >2) were subjected to an integrative analysis, using a Pearson correlation test (34 PTC evaluated by both analysis), aiming to obtain negative and positive significant correlations (*p* < 0.05).

**Fig. 1:**
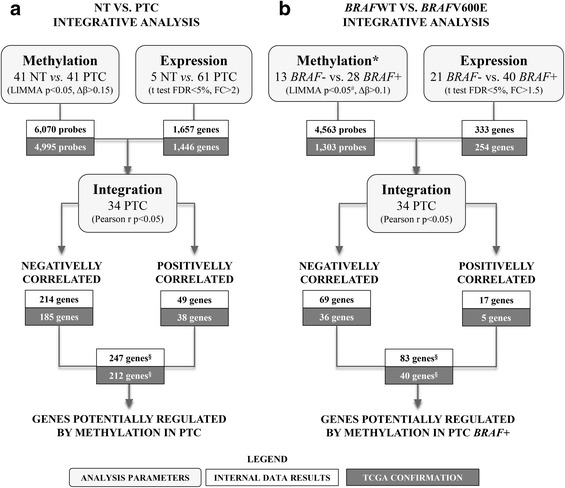
Workflow representative of the strategy used in the integrative analyses and in the cross-study validation. **a** Genome-wide methylation analysis revealed 6070 differentially methylated probes, and large-scale gene expression analysis identified 1657 differentially expressed genes in PTC (the last from a previous study). Corresponding probes/genes were submitted to a Pearson correlation test (34 PTC analyzed by both platforms) revealing 214 genes presenting probes with negative correlation and 49 genes with positive correlation. A total of 247 genes were classified as potentially regulated by DNA methylation in PTC. **b** A total of 4563 differentially methylated probes and 333 differentially expressed genes were identified in PTC according to *BRAF*V600E mutation. The Pearson correlation test revealed 69 and 17 genes with negative and positive correlation, respectively. Eighty three genes were classified as potentially regulated by DNA methylation in PTC *BRAF* mutated. *Tumor samples were initially corrected by NT samples (∆βPTC-∆βNT) and then *BRAF* positive and negative tumors were compared; ^§^Some genes presented both methylation probes negatively and positively correlated. ^#^Unadjusted *p* value

A cross-study validation was performed to confirm the results using DNA methylation microarray and RNA sequencing data from TCGA database (https://tcga-data.nci.nih.gov/docs/publications/tcga/). Similar parameters of the internal analysis were adopted to compare all conditions in the external dataset (*t* test *p* < 0.05, FDR <5% and Pearson correlation test *p* < 0.05) (details in Additional file 1). Figure 1 summarizes the strategy and results obtained in this analysis.

### In silico molecular interactions analysis

Disease and biological function and canonical pathway analysis including genes found in the integrative analysis were performed using Ingenuity pathway analysis (IPA-Ingenuity® Systems) and KEGG Orthology Based Annotation System (KOBAS—http://kobas.cbi.pku.edu.cn/) software version 2.0. Genes confirmed by TCGA database with negative correlation between expression and methylation probes were included to obtain a highly trustworthy analysis.

### Data confirmation by quantitative bisulfite pyrosequencing and RT-qPCR

In addition to the independent data confirmation using TCGA database, four genes (*ERBB3*, *FGF1*, *GABRB2*, and *HMGA2*) presenting methylation in the body gene region and two in the promoter regions (*FGFR2* and *RHD5*) were selected to be evaluated for quantitative bisulfite pyrosequencing and RT-qPCR analysis. The selection criteria of these genes are presented in Additional file 1.

The CpG methylation pattern was assessed by quantitative bisulfite pyrosequencing using the Pyromark Q96 ID system (Qiagen, Valencia, CA, USA) in samples microarray dependent (28 NT and 29 PTC) and independent (24 NT and 76 PTC). Gene expression analysis was performed by RT-qPCR in a 7500 Real Time PCR System (Applied Biosystems, Foster, CA, USA) in samples used in the microarray assays (4NT and 51 PTC) and in an independent set of cases (48NT and 54 PTC). The details of both procedures are presented in Additional file 1.

The pyrosequencing and RT-qPCR results were compared to tumor/normal status and according to clinical, pathological and genetic features by Student’s *t* test (*p* < 0.05) using GraphPad Prism 5.0 Software (Inc., La Jolla, CA, USA). Bonferroni correction was applied to adjust the *P* value by multiple hypotheses testing.

## Results

### DNA methylation and gene expression profiles in PTC

**Fig. 2:**
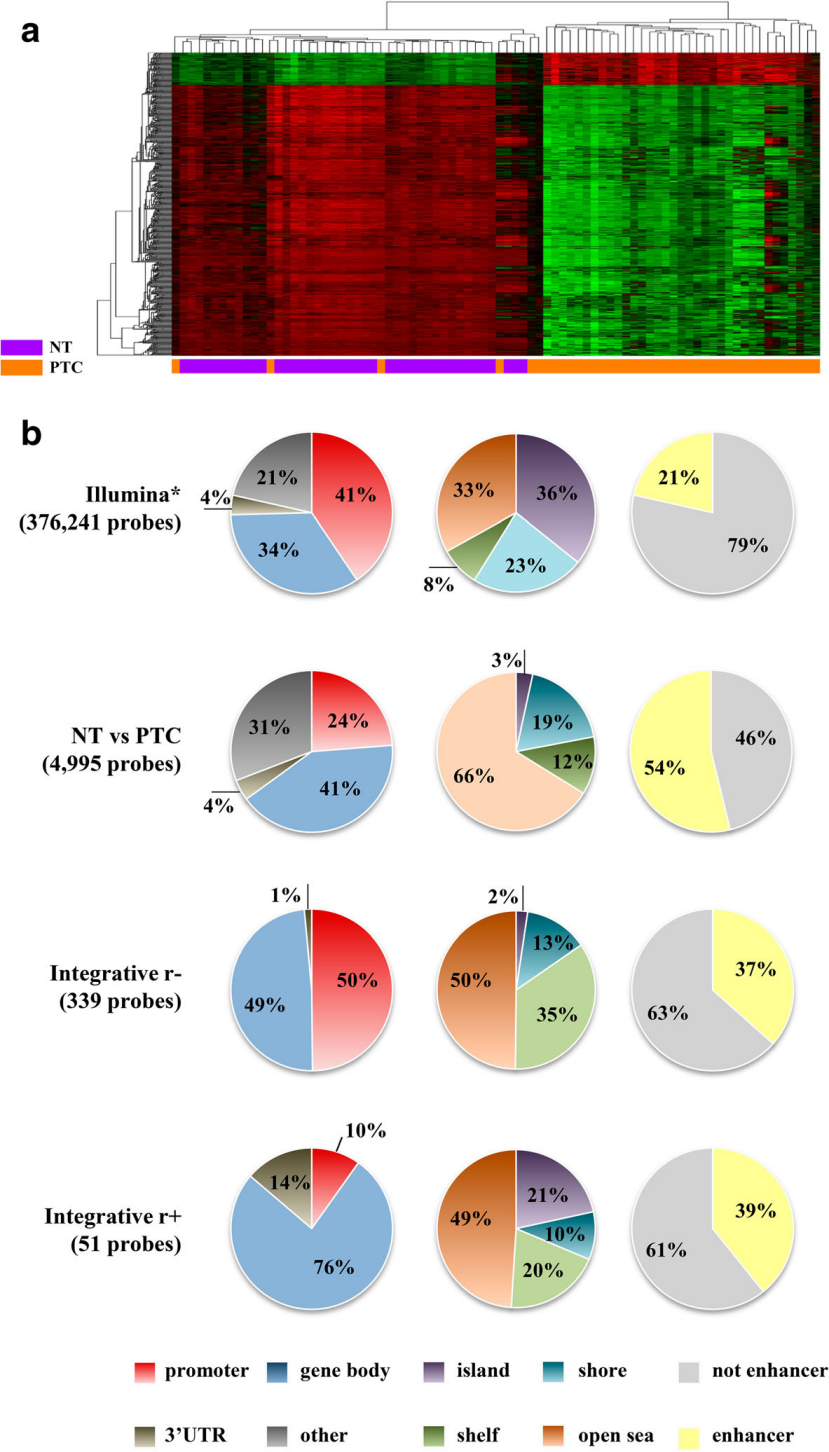
Classification of the differentially methylated probes in PTC. **a** Supervised hierarchical clustering analysis showed 6070 differentially methylated probes in papillary thyroid carcinoma (PTC) versus normal thyroid (NT) tissues, mostly hypomethylated in PTC. The first cluster shows all normal samples (*purple*) and six PTC (*orange*), and the second is composed exclusively by tumor samples (*orange*). The beta values vary between zero (*green*) and one (*red*). **b** Methylation probes identified in PTC versus NT and those detected in the integrative analysis with negative (r−) and positive correlation (r+) according to the functional genomic distribution, CpG content, and neighborhood context and enhancer representation

To identify differential methylation in PTC, we analyzed CpG methylation status comparing PTC with NT (*p* < 0.05; |Δβ| 0.15). This analysis revealed 6070 CpGs probes differentially methylated, of which 89% (5425) were hypomethylated. A supervised hierarchical clustering analysis revealed two main clusters, one comprised exclusively PTC samples and the other included all NT and six PTC cases (Fig. 2a).

A total of 4995 of 6070 available probes in the TCGA database was confirmed as differentially methylated (Additional file 2: Table S1, Additional file 3: Figure S1A), highlighting the robustness of our methylome analysis. An enrichment of the identified probes was detected in non-promoter regions (76 vs. 59% represented by the platform, *p* < 0.0001), mapped far from the CpG island called “open sea” (66 vs. 33% represented by the platform, *p* < 0.0001), and enhancers regions (54 vs. 21% represented by the platform, *p* < 0.0001) (Fig. 2b).

Using our previous genome-wide expression data [26] in 61 PTC versus five NT, 1657 differentially expressed genes were found (FDR <5% and FC >2). The comparison of these results with TCGA database, confirmed the involvement of 1446 genes differentially expressed (Additional file 4: Table S2, Additional file 3: Figure S1B).

### Integration of DNA methylation and gene expression profiles in PTC

A powerful tool used in the identification of novel driver alterations in cancer is the combined analysis of different molecular platforms (21). Accordingly, an integrative DNA methylation and gene expression analysis were performed, highlighting genes potentially regulated by DNA methylation. The methylation analysis revealed 867 probes representing 420 genes differentially expressed. A total of 214 and 49 genes were identified as negatively and positively correlated (185 and 38 confirmed in the TCGA), respectively, with the corresponding methylation probe (Additional file 5: Table S3). Curiously, about half of the negatively correlated probes (163) was found covering promoter regions and 2% were mapped in CpG islands, contrasting to only 10% (6) of the positively correlated probes in promoter regions (*p* < 0.0001) and 21% (11) in CpG islands (*p* < 0.0001). No differences were observed in the enhancer regions (*p* = 0.299) (Fig. 2b).

**Fig. 3:**
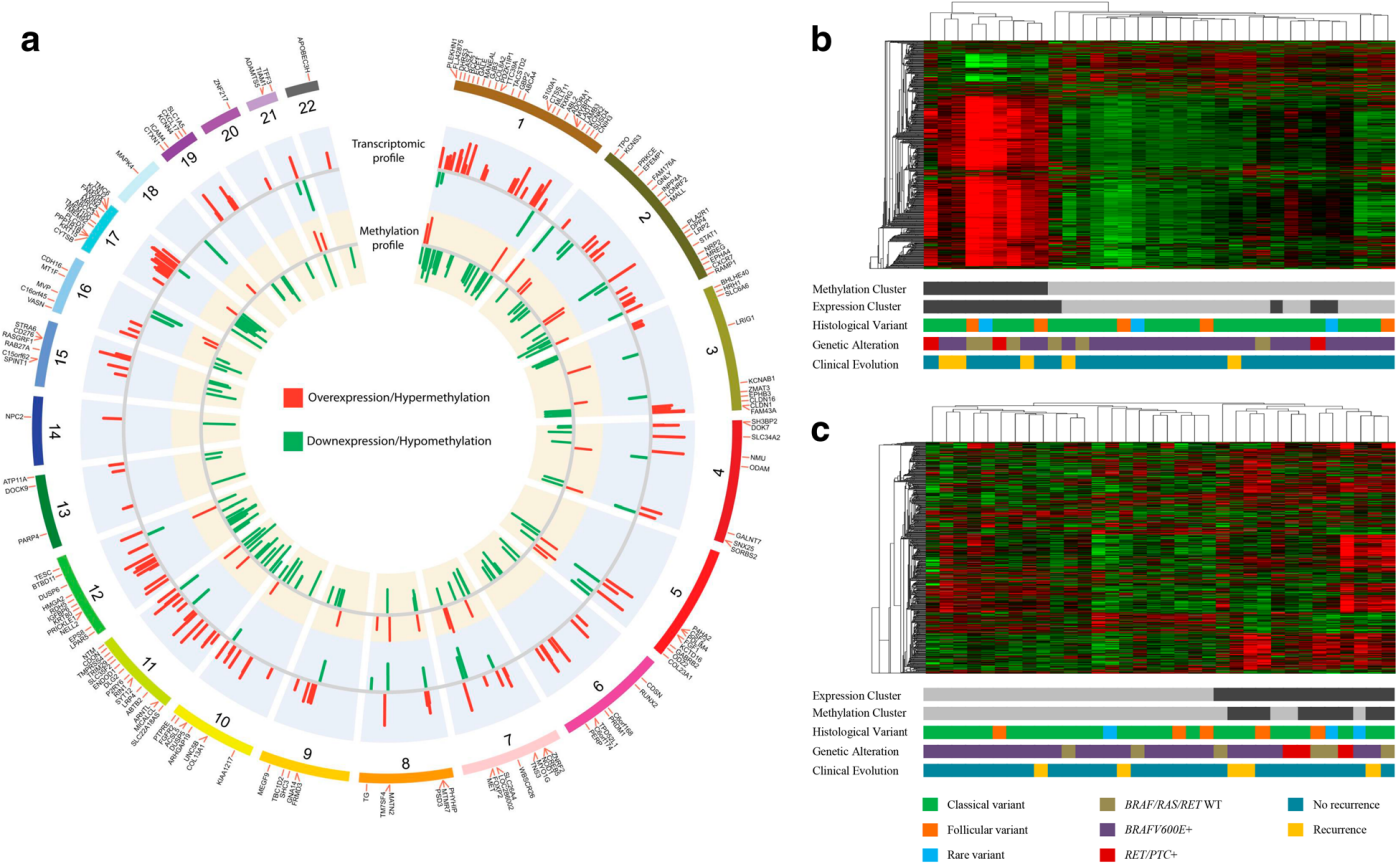
Methylation and gene expression profiling in PTC. **a** Genes identified in the integrative analysis with negative correlation and confirmed in the TCGA data. The outermost circle displays the human autosomal chromosomes, and the inner layers show both expression and methylation profiles. The figure was created following the parameters available in http://circos.ca. Unsupervised hierarchical clustering analysis revealed the **b** methylation and **c** gene expression profiles of 34 PTC evaluated with both platforms, and the relation with histological variant, genetic alteration, and follow-up. Two clusters were identified by both methodologies, and an overlapping between methylation and expression data was observed (*dark* and *gray* clustering). *Gray* cluster of methylation and gene expression was associated with a higher frequency of *BRAF*-mutated tumors (*p* = 0.034 and *p* = 0.013, respectively; Fisher’s exact test)

The genes uncovered by the integrative analysis (negatively correlated and confirmed as altered in the TCGA portal) were distributed through all autosomal chromosomes (Fig. 3a). Cellular movement, growth, proliferation, and survival were the most significant molecular functions (IPA software) involving these genes (Additional file 6: Table S4). The FGF (Additional file 3: Figure S2) and retinoic acid signaling (IPA and KOBAS 2.0, *p* < 0.01) were among the main canonical pathways involved in PTC (Additional file 6: Table S4).

### DNA methylation and gene expression profiling according to BRAF mutation

The *BRAF* (*BRAF*V600E) point mutation was detected in 28 of 41 PTC (68%) while no *RAS* (*HRAS*, *KRAS*, and *NRAS*) mutations were observed. The *RET* rearrangements (*RET/PTC1* and *RET/PTC3*) were found in five of 41 cases (12%). As expected, the alterations were mutually exclusive.

An unsupervised hierarchical clustering analysis, comprising 34 PTC evaluated by both methylation and expression arrays, revealed two distinct clusters (Fig. 3b, c). A substantial overlapping between methylation and expression clusters was observed. One cluster was enriched by *BRAF*V600E tumors in methylation and expression analysis (*p* = 0.034 and *p* = 0.013, respectively, Fisher’s exact test).

The importance of *BRAF* mutation in the methylation and expression profiles was evaluated using a similar approach described in the integrative analysis using PTC versus NT samples. A differential methylation profile (unadjusted *p* < 0.05; |Δβ| 0.1) was observed in PTC *BRAF* mutated (3312 hypomethylated and 1251 hypermethylated probes) compared with PTC *BRAF* wild-type (Additional file 7: Table S5). The expression profile unveiled 333 altered transcripts in *BRAF-*mutated tumors (FDR <5% and FC >1.5) (Additional file 8: Table S6).

The comparison with TCGA database showed similar methylation and gene expression pattern in 29 and 82% of the genes, respectively. Integrative analysis revealed 69 and 17 genes with significant negative and positive correlation (36 and 5 of them were also found in the TCGA), respectively (Additional file 9: Table S7).

### Validation of genes potentially regulated by methylation and association with clinical features

**Fig. 4:**
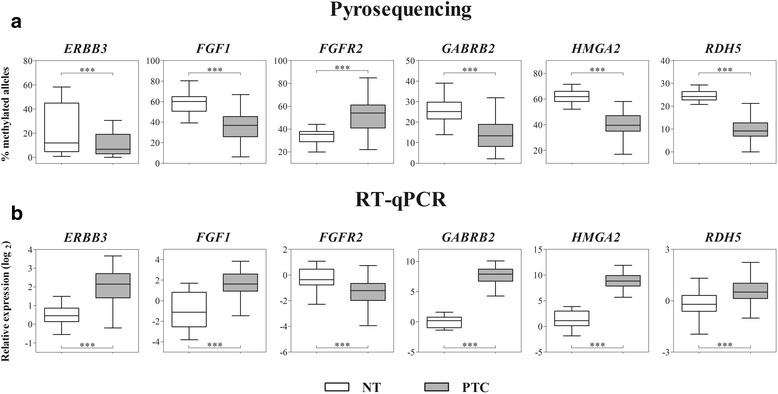
Methylation (**a**) and expression levels (**b**) confirmation of the selected genes. **a** *ERBB3*, *FGF1*, *GABRB2*, *HMGA2*, and *RDH5* hypomethylation and *FGFR2* hypermethylation were confirmed in PTC samples by pyrosequencing after DNA modification by bisulfite. **b**. *ERBB3*, *FGF1*, *GABRB2*, *HMGA2*, and *RDH5* overexpression and *FGFR2* downexpression were confirmed in PTC by RT-qPCR. The *boxplot* indicates the interquartile range and median. ***p < 0.001 by comparing PTC to NT (Student’s *t* test)

The genes investigated by pyrosequencing and RT-qPCR (selected from the integrative analysis and TCGA cross-study validation) were confirmed as differentially methylated/expressed, showing inverted methylation and expression patterns. *ERBB3* (*p* = 0.0005), *FGF1* (*p* < 0.0001), *GABRB2* (*p* < 0.0001), *HMGA2* (*p* < 0.0001), and *RDH5* (*p* < 0.0001) genes were hypomethylated and over-expressed (*p* < 0.0001). Moreover, *FGFR2* was hypermethylated and downexpressed (*p* < 0.0001 and *p* < 0.0001, respectively) (Fig. 4a, b). Increased expression of *ERBB3* (*p* < 0.0001) and *GABRB2* (*p* < 0.011) and hypomethylation of *FGF1* (*p* = 0.0003), *GABRB2* (*p* < 0.014), and *RDH5* (*p* < 0.016) were significantly associated with *BRAF*-mutated tumors (Additional file 3: Figure S3). No significant association was detected in the comparison between clinical parameters and the markers evaluated by both RT-qPCR and pyrosequencing (Bonferroni-adjusted *p* > 0.05) (Additional file 10: Table S8).

## Discussion

The purpose of this study was to characterize the DNA methylation pattern of PTC and to elucidate its effect on gene expression deregulation and the biological pathways associated with the disease. By comparing PTC with matched NT, a global hypomethylation was detected, as previously reported [19, 20, 23]. Loss of DNA methylation throughout the genome has been related to genomic instability, somatic driver mutations, chromosomal breaks, and rearrangements. Papillary thyroid tumors have been characterized by a low number of structural rearrangements and frequent somatic driver mutations [17, 19, 20, 23].

CpG islands (CGI) are well described in literature as often found in promoters and associated with gene downregulation when hypermethylated [25]. In our study, half of the methylated probes presenting inverted patterns compared with gene expression (negative correlation) were annotated in promoters, contrasting with only 10% of probes showing positive correlation. Recently, epigenetic studies have uncovered that methylation alterations at other regulatory regions such as CGI shores, non-CGI promoter, and enhancers might also play a role in tumorigenesis [17–23, 25]. In our study, these regions were more frequently detected, agreeing with previous reports in PTC samples [20].

Gene bodies are reported as having a limited number of CpG islands and broadly methylated. Interestingly, this region harbors multiple repetitive and transposable elements [25]. However, the biological significance of DNA methylation in this region is poorly clarified. Gene body methylation does not necessarily block the transcription as observed in promoter regions, but might increase the transcriptional activity, stimulate the transcription elongation, and impact in the splicing [25, 29]. Accordingly, 80% of the probes presenting positive correlation between methylation/expression were annotated in gene body or 3’UTR. Interestingly, differentially methylated probes exclusively mapped in body or in promoter regions presented a similar proportion of differentially expressed genes (14 and 18%, respectively). Furthermore, an opposite relation between methylation and expression (hypomethylation/overexpression or hypermethylation /downexpression) of probes mapped exclusively in the promoter or in body gene regions were also detected (73 and 93%, respectively). These findings suggest that body gene methylation is a process involved in gene expression regulation, similar to those described in the promoter regions.

From 1446 genes differentially expressed (internal and external data), only 212 were considered as regulated by DNA methylation, suggesting the involvement of other transcription-regulator mechanisms. Nonetheless, one of the most significant results herein described was the enrichment of methylation disruption in enhancer regions (54% of the methylated probes). Enhancers are non-coding regulatory sequences able to recruit transcription factors and activate promoters. These regions are located from a few kilobases to more than a megabase of the transcription start site of the target gene [30]. Although the exact mechanism of the regulatory proteins binding according to DNA methylation in enhancers is still unclear, hypomethylation seems to result in increased activity of the target gene [31]. Therefore, even if only a few genes were directly associated with the regulation of DNA methylation, several genes might be influenced by the altered methylation in enhancer regions. Aran and Hellman [32] suggested that gene expression variation could be explained by methylation in enhancers.

The in silico analysis comprising 185 genes found in the integrative analysis (inverted methylation/expression pattern and confirmed by the cross-study validation) highlighted the importance of epigenetic alterations in thyroid carcinogenesis. The predicted effects in the biological functions (cellular movement, growth, proliferation, and survival) were previously reported as associated with thyroid cancer development [33]. Furthermore, deregulated canonical pathways including the retinoic acid- and FGF signaling pathways were unveiled as associated to PTC. The retinoic acid (RA) is involved in cell differentiation and plays a fundamental role in preventing neoplastic growth [34]. The RA biosynthesis involves the RDH5 enzyme, which reversibly oxidizes all-trans-retinol to all-trans-retinaldehyde and them irreversibly oxidized to RA by retinoid-active aldehyde enzymes (ALDH1A) [35, 36]. We found *RDH5* hypomethylation and overexpression and *ALDH1A1* downexpression in PTC, suggesting a dysregulation of RA metabolism. Retinoic acid has been implicated in re-differentiation of thyroid cells by the induction of sodium-iodide symporter (*NIS*) expression, which is responsible for the iodine internalization [37, 38]. Loss of *NIS* expression has been associated to a low uptake of iodine and interference in the efficacy of the radioiodine therapy in thyroid tumors [39]. According to our results, the methylation changes are potentially involved in the dedifferentiation of PTC cells, as a result of RA signaling pathway disruption.

Fibroblast growth factor signaling pathway, involved in angiogenesis and tumorigenesis [40], was significantly altered in our PTC samples. *FGF1* and *FGF2* have been reported as over-expressed in differentiated thyroid tumors, but their receptors present contrasting results [41–43]. In our study, *FGF1* was found hypomethylated and over-expressed and *FGFR2* was hypermethylated and downexpressed. According to Kondo et al, *FGFR2* hypermethylation promotes downexpression and its re-expression acts blocking the BRAF/MAPK pathway in thyroid cancer [44].

Similar to the literature, mutually exclusive somatic alterations were found in *BRAF* (68% of PTC samples) and *RET* (12%), while *RAS* had no mutations [18, 45, 46]. The supervised clustering analysis of the methylation profiling revealed six PTC grouped with NT samples, two of them were *BRAF*V600E and one *RET-PTC*3 positive. The inclusion of PTC samples in NT enriched clusters was previously reported in methylation profiling studies [19]. This finding could be explained by non-tumor cells contamination, as also observed in the TCGA study [21].

The hierarchical clustering analysis showed a substantial overlapping between transcripts and methylation profiles. A cluster enriched with of *BRAF*V600E tumors was detected, in agreement with previous studies using methylation [20] and gene expression analysis [45]. Kikuchi et al [18] evaluated the methylation profile (Infinium HumanMethylation27K Illumina) of 14 PTC and 10 normal thyroid tissues. Among the 25 differentially methylated genes, six (*HIST1H3J*, *POU4F2*, *SHOX2*, *PHKG2*, *TLX3*, and *HOXA7*) were selected for data confirmation. The authors described hypermethylation and downexpression of these genes in an additional set of cases and an association with *BRAF/RAS* mutations. From these genes, only *PHKG2* was found hypomethylated (promoter region) of our gene list. The supervised analysis revealed an even more evident hypomethylated state in tumors harboring *BRAF*V600E, where 73% of the probes were less methylated compared to wild-type *BRAF* tumors (95% considering the TCGA confirmed probes). The integrative analysis according to *BRAF* mutation uncovered 69 genes showing negative correlation, 36 of them were confirmed by external data (TCGA). Similarly, Mancikova et al [19] reported genes with inverse correlation between methylation and gene expression mainly associated to MAPK pathway (*MAPK13*, *DUSP5*, and *RAP1GA1)* and apoptosis (*LCN2*, *RIPK1*, and *LGALS1*). The stratification of PTC into two entities, the *BRAF-like* (classical or tall variant) and *RAS-like* (composed mainly by the follicular variant), based on molecular landscape was generated from *omics* integrative analysis in a cohort of 496 PTCs [21]. Considering only the DNA methylation levels, the authors described four groups, two of them enriched by *H/K/NRAS*-mutated follicular variant PTC (follicular and CpG island methylated) and two enriched by *BRAF*-mutated classical and tall cell PTC (classical 1 and classical 2). Similar to our findings, composed largely by *BRAF*-mutated tumors, the TCGA classical/tall cell PTC-enriched cluster was distinguished by low levels of methylation in CpG normally methylated outside of islands [21].

In addition to RDH5 (RA pathway), FGF1, and FGFR2 (both from FGF signaling pathway), ERBB3, GABRB2, and HMGA2 were previously reported as over-expressed in PTC [43–49]. Methylation and expression pattern of these genes were also confirmed by pyrosequencing and RT-qPCR. Previously, we pointed out GABRB2 and HMGA2 as potential diagnostic makers in thyroid tumors [26]. HMGA2 interacts with the transcription machinery (acts in the chromatin structure and regulates the transcription) and in the epithelial-mesenchymal transition (by repression of E-cadherin) [47, 50, 51]. Although *HMGA2* and *GABRB2* were not associated with clinical features or somatic alterations in the large-scale expression analysis, the independent validation demonstrated that *GABRB2* hypomethylation and overexpression were significantly altered in *BRAF*V600E tumors. Tumors harboring this mutation were associated with *ERBB3* overexpression [45, 48] but not with DNA methylation. A plausible explanation is the involvement of different mechanisms associated with the *ERBB3* regulation in *BRAF* tumors, including the involvement of miRNAs or other post-transcription regulation events [49, 52]. Overexpression and oncogenic activation of *ERBB3* have been associated to treatment resistance with RAF/MEK inhibitors in melanoma and thyroid cancer, especially in the specific context of *BRAF*V600E [53–55].

## Conclusions

Papillary thyroid cancer was largely characterized by methylation loss, mainly in *BRAF*V600E PTC. The alterations were distributed throughout the genome, albeit overrepresented by enhancers. Promoter region deregulations were related to an inverse pattern of gene expression levels as expected, while non-promoter regions consisted by both negative and positive correlations. The integrative analysis combined with a cross-study validation allowed the identification of genes acting in essential pathways associated with PTC pathogenesis and progression. Furthermore, potential drivers, therapeutic targets, and biomarkers are highlighted, which could be useful in the management of PTC patients.

## Additional files

Additional file 1: Material and methods. (DOCX 52 kb)

Additional file 2: Table S1. Methylation data analysis in our cohort of PTC samples and in the TCGA database. Legend. *FGD* functional genomic distribution, *CHR* chromosome, *DMR* differential methylation region, *RDMR* reprogramming-specific differentially methylated region, *CDMR* cancer-specific differentially methylated region, *ND* not described. (XLSX 928 kb)

Additional file 3: Figure S1. In silico validation from (A) methylation and (B) expression analysis comparing with The Cancer Genomes Atlas (TCGA) database. **Figure S2.** FGF canonical signaling pathway potentially activated in PTC. Dark green molecules indicate genes downexpressed and hypermethylated in PTC samples. Dark red molecules indicate upregulated and hypomethylated genes. Light red molecules represent overexpression, and light green molecules represent downexpression. Both colors in the same molecules indicate different members of the same family with contrary expression levels. **Figure S3.** (A) DNA methylation and (B) gene expression levels detected in the selected genes according to *BRAF*V600E mutation. A. *FGF1*, *GABRB2* and *RDH5* hypomethylation were associated with *BRAF*V600E PTC samples by pyrosequencing. B. *ERBB3* and *GABRB2* overexpression were associated to *BRAF*V600E PTC samples by RT-qPCR. (DOCX 4545 kb)

Additional file 4: Table S2. Expression analysis of PTC versus NT and comparison with TCGA database portal. Legend. *FC* fold change, *FDR* false discovery rate, *NS* not significant, *NA* not available. (XLSX 115 kb)

Additional file 5: Table S3. Integrative analysis between methylation and expression data in PTC samples. Legend. *FGD* functional genomic distribution, *CHR* chromosome, *DMR* differential methylation region, *RDMR* reprogramming-specific differentially methylated region, *CDMR* cancer-specific differentially methylated region, *NA* not available, *NS* not significant, *FC* fold change, *FDR* false discovery rate, *R* inverse correlation. (XLSX 122 kb)

Additional file 6: Table S4. Disease, biological functions, and canonical pathways potentially regulated by methylation in PTC samples. (XLSX 11 kb)

Additional file 7: Table S5. Differentially methylated probes in *BRAF*V600E PTC samples. Legend. *FGD* functional genomic distribution, *CHR* chromosome, *DMR* differential methylation region, *RDMR* reprogramming-specific differentially methylated region, *CDMR* cancer-specific differentially methylated region, *ND* not described, *NA* not available, *NS* not significant. (XLSX 624 kb)

Additional file 8: Table S6. Expression data analysis in PTC versus NT samples and comparison with TCGA data according to the *BRAF* mutation. Legend. *FC* fold change, *FDR* false discovery rate. (XLSX 30 kb)

Additional file 9: Table S7. Integrative analysis between methylation and expression data according to the *BRAF* mutation. Legend. *FGD* functional genomic distribution, *CHR* chromosome, *DMR* differential methylation region, *RDMR* reprogramming-specific differentially methylated region, *CDMR* cancer-specific differentially methylated region, *NA* not available, *NS* not significant, *FC* fold change, *FDR* false discovery rate, *R* inverse correlation. (XLSX 39 kb)

Additional file 10: Table S8 .Association between the markers selected for validation and clinical features. Legend. *FC* fold change of PTC/NT, *Delta* pyrosequencing methylation percentage of PTC minus NT, *ETE* extrathyroidal extension; in bold: significant *p* value (*t* test <0.05); bold and underline: significant *p* value after Bonferroni correction (*t* test *p* < 0.0042). (XLSX 12 kb)

## Abbreviations

CGI: CpG islands
FGF: Fibroblast growth factor
IPA: Ingenuity pathways analysis
KOBAS: KEEG orthology based annotation system
MAPK: Mitogen-activated protein kinase
NT: Non-neoplastic adjacent tissues
PI3K: Phosphatidylinositol 3-kinase
PTC: Papillary thyroid carcinoma
RA: Retinoic acid
TCGA: The Cancer Genome Atlas
TSHR: Thyroid gland function.
