# Supplementary material for: Integrated data analysis reveals potential drivers and pathways disrupted by DNA methylation in papillary thyroid carcinomas
Source: Clin Epigenetics. 2017 May 2;9:45. doi: 10.1186/s13148-017-0346-2 (PMC5414166; doi:10.1186/s13148-017-0346-2)
Supplement: Supplementary file 1 — Material and methods. (DOCX 52 kb) [file 13148_2017_346_MOESM1_ESM.docx]

**Integrated data analysis reveals distinct drivers and pathways disrupted by DNA methylation in papillary thyroid carcinomas**

Caroline Moraes Beltrami^1^, Mariana Bisarro dos Reis^1,2^, Mateus Camargo Barros-Filho^1^, Fabio Albuquerque Marchi^1^, Hellen Kuasne^1,5^, Clóvis Antônio Lopes Pinto^3^, Srikant Ambatipudi^4^, Zdenko Herceg^4^ , Luiz Paulo Kowalski^1,5^, Silvia Regina Rogatto^1,6^*

**SUPPLEMENTARY METHODS**

*Detection of BRAF, KRAS, HRAS and NRAS mutation*

Forward and reverse biotinylated primers (Epigendx, Worcester MA, UK) (0.2μM), 15μL of *MasterMix* (Qiagen, Valencia, CA, USA), 20ng of DNA sample and nuclease-free water were added to a final volume of 30μL. The PCR cycling conditions were 95ºC for 15 min, followed by 45 cycles of 95ºC for 30 seconds, specific annealing temperature (58ºC for *BRAF* and *NRAS*, 62ºC for *HRAS* and 64ºC for *KRAS*) for 30 seconds and 72ºC for 30 seconds, followed by a final step of 72ºC during 10 min. Negative and positive-mutation controls and no template control (NTC) were included in the reactions. The amplicon was confirmed in agarose gel (1%) stained with *SYBR Safe DNA Gel Stain* (0.55μg/mL) (Invitrogen, Eugene, Oregon, USA). The supplementary Table M1 shows the primer sequences and the alteration evaluated for each gene investigated.

The pyrosequencing results were evaluated according to the quality of the fluorescence peak and interpreted according to the presence or absence of the mutations. Cases with more than 10% of alleles altered were considered as mutated.

*RET/PTC1 and RET/PTC3 rearrangements*

The *RET*/PTC1 and *RET*/PTC3 inversions detection were evaluated according to Rhoden et al [1] using Taqman assays (*Applied Biosystems,* Warrington, UK) (Supplementary Table M2).

The amplification reaction (PCR 7500 Real Time PCR System, Applied Biosystems) was performed using 50-100nM of probes, 200-250nM of each primer sequences (Applied Biosystems), Taqman *Universal PCR Master Mix* (Applied Biosystems), 20ng of cDNA and nuclease-free water in a total volume of 20µL for 40 cycles. Technical replicates, positive-controls of the mutations and a reference (no cDNA) were included in all assays. The normalization of the quantification-cycle values was performed according to Pfaffl [2], including an endogenous-control (*ACTB*). *RET*/PTC1 or *RET*/PTC3 expression was considered positive with Cq < 35 with positive *ACTB* expression (Cq<30).

*DNA treatment with sodium bisulfite*

Genomic DNA was bisulfite-modified using EZ DNA Methylation Gold kit ~~D5006~~ (Zymo Research, Irvine, CA, USA) according to the Illumina recommendations (16 cycles at 95ºC for 30 seconds, 50ºC for 1 hour and 4ºC for 10 min). Methylation levels of specific targets were evaluated by pyrosequencing method using bisulfite modification according to the manufacturer’s protocol.

*Genome wide DNA methylation data processing*

Probes overlapping known SNPs, cross-reactive and mapped on the X and Y chromosomes were removed from the preprocessing step. The methylation data were analyzed using β values [3]. Probes were filtered for low bead count (<3) and low quality (p detection >0.05) using WateRmelon package. Samples having 1% of sites with a detection p-value > 0.05 were removed. Data was adjusted for color bias, inter-sample normalization and corrected using BMIQ normalization [4]. A linear regression analysis was performed adjusting data for gender and age. The batch effects were corrected using sva package [5].

*Cross-Study Validation*

Genome-wide methylation (PTC *versus* NT) data were collected from TCGA public database (June, 2016). DNA methylation (β values) using HumanMethylation450 platform (level 3) was obtained from the “THCA - Thyroid carcinoma” (https://tcga-data.nci.nih.gov/tcga/). Similar approach was performed for RNA sequencing using Illumina HiSeq 2000 platform (level 3) data. The methylation analysis was carried out using matched 56 NT/PTC samples (pairs). The gene expression analysis included 59 NT and 506 PTC. T test was applied to compare all conditions (paired in methylation and unpaired in gene expression analysis) using BRB array tool software (P<0.05 and FDR<5%). A Pearson correlation test was further applied to contrast methylation and gene expression from 506 PTC samples evaluated by both platforms (P<0.05). *BRAF* mutation status in tumor samples was verified by cBio Cancer Genomics Portal (<http://www.cbioportal.org/>). Similar cross-study validation was performed according to *BRAF* mutation status.

*Pyrosequencing and Reverse Transcription Quantitative PCR assays (RT-qPCR)*

Pyrosequencing and RT-qPCR were performed in six genes selected from the integrative analysis, presenting negative correlation and validated in the cross-study validation using TCGA data. *GABRB2* and *HMGA2* were selected based on gene expression analysis (first and second highest FC, respectively; both in body gene), *RDH5* on methylation (promoter region and fourth highest delta beta), integrative analysis (highest negative correlation) and pathway analysis (retinoic acid related pathways). *FGF1* and *FGFR*2 were included based on pathway analysis (body gene and promoter region, respectively; key members of the FGF signaling) and *ERBB3* from the integrative analysis (body gene; 22^nd^ highest negative correlation) and literature reports of thyroid cancer.

*Pyrosequencing reaction.* The primers were designed according to the following criteria: (a) PCR primers size between 18 and 24 base pairs (bp), (b) three G (guanine) or C (cytosine) at 5’end of the sequences were avoided, (c) similar quantity of C and G in forward and reverse primers, (d) at least 50% of C and G content, (e) amplicon up to 400bp, and (f) melting temperature between 55 and 62ºC. Stable secondary structures and the complementarity between 5’ and 3’end were avoided. The primer sequences for specific target and annealing temperature used in the PCR are detailed in Supplementary Table M3.

After treatment with sodium bisulfite, 25ng of each DNA-modified sample were amplified by PCR. The PCR amplification was performed using the PyroMark PCR kit (Qiagen, Valencia, CA, USA) in a final volume of 30 µL according to the manufacturer recommendations. The PCR cycling conditions used were 95ºC for 15 minutes, 50 cycles (95ºC for 30 seconds, annealing temperature for 30 seconds and 72ºC for 1 min and 10 seconds) and a final step of 72ºC for 10 min. Human HCT116 DKO Methylated DNA and unmethylated control (Human HCT116 DKO Non-Methylated DNA (Zymo Research, Irvine, CA, USA) were used in each assay.

*Reverse Transcription Quantitative PCR assay (RT-qPCR).* RNA was isolated using TRIZOL™ (Invitrogen, Carlsbad, CA, USA) or RNeasy mini kit (Qiagen, Valencia, USA), according to the manufacturer’s instructions. RNA integrity was verified using the Agilent 2100 Bioanalyzer RNA 6000 LabChip kit (Agilent Technologies, Santa Clara, CA, USA). Total RNA samples (RIN>5) were treated with DNAse I (Invitrogen, Carlsbad, CA, USA). The reverse transcription (cDNA) was performed using the SuperScript III Reverse Transcriptase (Invitrogen, Carlsbad, CA, USA). The oligo dT12-18 (Invitrogen) and random hexamers primers (Invitrogen) were used for cDNA synthesis.

Quantitative RT-qPCR was performed using the TaqMan® Gene Expression Assays (Applied Biosystems, Warrington, UK) to evaluate *ERBB3*, *GABRB2* and *HMGA2* genes and *EIF2B1* and *PUM1* as reference transcripts, as previously reported [6]. The Supplementary Table M4 shows the primer sequences used for the selected set of genes investigated by RT-qPCR. The SYBR® Green detection assay (Applied Biosystems, Warrignton, UK) was applied to evaluate the expression levels of *FGF1*, *FGFR2* and *RDH5* genes and *GUSB* and *HPRT* as reference transcripts*.* Duplicate reactions were performed using 1X SYBR® Green I Master Mix, 200µM of forward and reverse primers and 10ng of cDNA huddled with robot pipetting QIAgility (Qiagen, Courtaboeuf, France) in a total volume of 12.5 µL. The amplifications were carried out in a 7900HT Real Time PCR System (Applied Biosystems). The PCR cycling conditions consisted of 50ºC for 2 minutes, 95ºC for 10 minutes and a final step of 40 cycles (95ºC for 15 seconds and 60ºC for 1 min). Relative expression was calculated according to Pfaffl (2001). The analysis followed the guideline described by Bustin et al [7].

**REFERENCES**

1. Rhoden KJ, Johnson C, Brandao G, Howe JG, Smith BR, Tallini G. Real-time quantitative RT-PCR identifies distinct c-RET, RET/PTC1 and RET/PTC3 expression patterns in papillary thyroid carcinoma. Lab Invest. 2004;84:1557-1570.

2. Pfaffl MV. A new mathematical model for relative quantification in real-time RT-PCR. Nucleic Acids Res. 2001;29: e45.

3. Chen YA, Lemire M, Choufani S, Butcher DT, Grafodatskaya D, Zanke BW, et al. Discovery of cross-reactive probes and polymorphic CpGs in the Illumina Infinium Human Methylation 450 microarray. Epigenetics. 2013;8:203-209.

4. Teschendorff AE, Marabita F, Lechner M, Bartlett T, Tegner J, Gomez-Cabrero D, et al. A beta-mixture quantile normalization method for correcting probe design bias in Illumina Infinium 450 k DNA methylation data. Bioinformatics. 2013;29:189-196.

5. Leek JT, Johnson WE, Parker HS, Jaffe AE, Storey JD. The sva package for removing batch effects and other unwanted variation in high-throughput experiments. Bioinformatics. 2012;28:882-883.

6. Barros-Filho MC, Marchi FA, Pinto CA, Rogatto SR, Kowalski LP. High Diagnostic Accuracy Based on CLDN10, HMGA2, and LAMB3 Transcripts in Papillary Thyroid Carcinoma. J Clin Endocrinol Metab. 2015;100:E890-9.

7. Bustin SA, Benes V, Garson JA, Hellemans J, Huggett J, Kubista M, et al. The MIQE guidelines: minimum information for publication of quantitative real-time PCR experiments. Clin Chem. 2009;55:611-622.

**Supplementary Table M1.** Primer sequences used in the pyrosequencing reaction to identify *BRAF* and *RAS* mutations.

| Gene/  Codon | Primers | Sequence | Alteration |
| --- | --- | --- | --- |
| **BRAF/600** | ADS871 | TACA**GTG** | GTG → GAG |
| **KRAS/12,13** | ADS747 | **GTGGC**GTAGGCAA | GGT GGC → GAT GAT; AGT GAT; GCT GAT; GTT GAT |
| **HRAS/61** | ADS2293 | **CAG**GAGGAG | CAG → CTG |
| **NRAS/61** | ADS214 | GCTGGA**CA** | CAA → CGA or CTA or CCA |

The nucleotides tested for each gene are underlined,

**Supplementary Table M2.** Primers and probes used to investigate the *RET*/PTC inversion.

| Gene | Primer sequences | Amplicon |
| --- | --- | --- |
| **ACTB*** | F - 5'AGCCTCGCCTTTGCCGA3'  R - 5'CTGGTGCCTGGGGCG3'  VIC-CCGGCTTCGCGGGCGAC-TAMRA | 115 bp |
| **RET^#^** | F - 5'TGCTTCTGCGAGCCCG3'  R - 5'ATCACCGTGCGGCACAG3'  FAM-CATCCAGGATCCACTGTGCGACGA-TAMRA | 62 bp |
| **RET/ PTC1** | F – 5'CGCGACCTGCGCAAA3'  R - 5'CAAGTTCTTCCGAGGGAATTCC3’  FAM-CAAGCGTAACCATCGAGGATCCAAAGT-TAMRA | 66 bp |
| **RET/ PTC3** | F - 5'CCCCAGGACTGGCTTACCC3'  R - 5'CAAGTTCTTCCGAGGGAATTCC3'  FAM-AAAGCAGACCTTGGAGAACAGTCAGGAGG-TAMRA | 81 bp |

F: forward primer; R: reverse primer; bp: base-pairs; *Reference transcript; ^#^Wild-type transcript; [ ] concentration.

**Supplementary Table M3.** Primer sequences and properties of the genes selected for data confirmation evaluated by pyrosequencing.

| **Gene** | **Sequence** | **Product Length** | **AT** | **Number of CpG sites** |
| --- | --- | --- | --- | --- |
| ***ERBB3*** | F – 5’ GTAGGAGGTTTAATAGAGGTTGG 3’ | 389bp | 56°C | 1 |
|  | R – 5’ [B]ATCCCAAAAACACCCCAAAA 3’ |  |  |  |
|  | Seq - 5’ GTAGGAGGTTTAATAGAGGTTGG 3’ |  |  |  |
|  | Py – 5’ **CG**TT 3' |  |  |  |
| ***FGF1*** | F - 5´ GGTTGGTTTTTTTGTAGAAGTGG 3´ | 370bp | 55°C | 1 |
|  | R - 5´ [B]AACCCCAAATCACACTACAA 3´ |  |  |  |
|  | Seq – 5’ AAGAGTTTGGTTAGT 3' |  |  |  |
|  | Py – 5’ **CG**GTT 3' |  |  |  |
| ***FGFR2*** | F – 5’ GGTTTATAGAAATGTGTGAGG 3' | 293bp | 50°C | 1 |
|  | R – 5’ [B]AACAAACTCCTCCAACAA 3' |  |  |  |
|  | Seq – 5' TGGTATAATTAATTT 3' |  |  |  |
|  | Py – 5’ **CG**TT 3' |  |  |  |
| ***GABRB2*** | F – 5’ AGTAGGTAGGTATAGGGGGT 3’ | 355bp | 49°C | 4 |
|  | R – 5’ [B]CTCTCTTCCCTCTCCCTAAT 3’ |  |  |  |
|  | Seq – 5’ TTAAAAAATGTTAA 3' |  |  |  |
|  | Py –5’ **CG**GTGGTTTTT**CG**TAAGT**CG**GGTTTTTT**CG**G |  |  |  |
| ***HMGA2*** | F – 5’ GATGTGTTTATTGGTGTTAGTGG 3’ | 461bp | 56°C | 3 |
|  | R – 5’ [B]TACTTCAATTCTCCACTTCCTT 3’ |  |  |  |
|  | Seq – 5’ TTAAATGGGATG 3’ |  |  |  |
|  | Py – 5’ **CG**AATTTTTATTGAGGA**CG**TTTATGGGTTTTA AGTTAATTAGTTTTATTTAATTAT**CG**A 3’ |  |  |  |
| ***RDH5*** | F - 5´ TTAAGGGGTGGGGGGAGTTT 3´ | 157bp | 55°C | 1 |
|  | R - 5´ [B]TCCCAACCTCCCACACTAAC 3´ |  |  |  |
|  | Seq - 5´ ATATATTTAATA 3´ |  |  |  |
|  | Py - 5´ **CG**TTTGG 3´ |  |  |  |

F: Forward; R: Reverse; Seq: primer used in the sequencing reaction; bp: base pairs; AT: Annealing temperature. Py: Reference sequence. CGs indicating the CpG analyzed are underlined.

**Supplementary Table M4.** Primer sequences and properties of the genes evaluated by RT-qPCR.

| **Detection Method** | **Gene** | **Sequence** | **Size (bp)** | **AT** |
| --- | --- | --- | --- | --- |
| **SYBR® GREEN** | *FGF1* | F – 5’ ACGGCTCACAGACACCAAAT 3' | 270 | 60°C |
|  |  | R – 5’ TTTGGGTCAACCAGGTGAGG 3' |  |  |
|  | *FGFR2* | F – 5’ TCTGGTCCTTCGGGGTGTTA 3' | 194 | 60°C |
|  |  | R – 5’ GAACGTTGGTCTCTGGGAGG 3' |  |  |
|  | *RDH5* | F - 5´ GCCTGCACAAAAATAAGCAC 3´ | 95 | 60°C |
|  |  | R - 5´ TAAATGGGGTCAGCCTTCCA 3´ |  |  |
| **TAQMAN®** | **Gene** | **ASSAY** | **Sise (pb)** | **AT** |
|  | *ERBB3* | Hs00951456_g1 | 81 | 60°C |
|  | *GABRB2* | Hs00241451_m1 | 143 | 60°C |
|  | *HMGA2* | Hs00971724_m1 | 81 | 60°C |

F: Forward; R: Reverse; bp: base pairs; AT: annealing temperature.
