# Supplementary material for: Integrated data analysis reveals potential drivers and pathways disrupted by DNA methylation in papillary thyroid carcinomas
Source: Clin Epigenetics. 2017 May 2;9:45. doi: 10.1186/s13148-017-0346-2 (PMC5414166; doi:10.1186/s13148-017-0346-2)
Supplement: Supplementary file 3 — In silico validation from (A) methylation and (B) expression analysis comparing with The Cancer Genomes Atlas (TCGA) database. Figure S2. FGF canonical signaling pathway potentially activated in PTC. Dark green molecules indicate genes downexpressed and hypermethylated in PTC samples. Dark red molecules indicate upregulated and hypomethylated genes. Light red molecules represent overexpression, and light green molecules represent downexpression. Both colors in the same molecules indicate different members of the same family with contrary expression levels. Figure S3. (A) DNA methylation and (B) gene expression levels detected in the selected genes according to BRAFV600E mutation. A. FGF1, GABRB2 and RDH5 hypomethylation were associated with BRAFV600E PTC samples by pyrosequencing. B. ERBB3 and GABRB2 overexpression were associated to BRAFV600E PTC samples by RT-qPCR. (DOCX 4545 kb) [file 13148_2017_346_MOESM3_ESM.docx]

**SUPPLEMENTARY FIGURES**

**Supplementary Figure 1.** *In silico* validation from **(A)** methylation and **(B)** expression analysis comparing with The Cancer Genomes Atlas (TCGA) database.

**
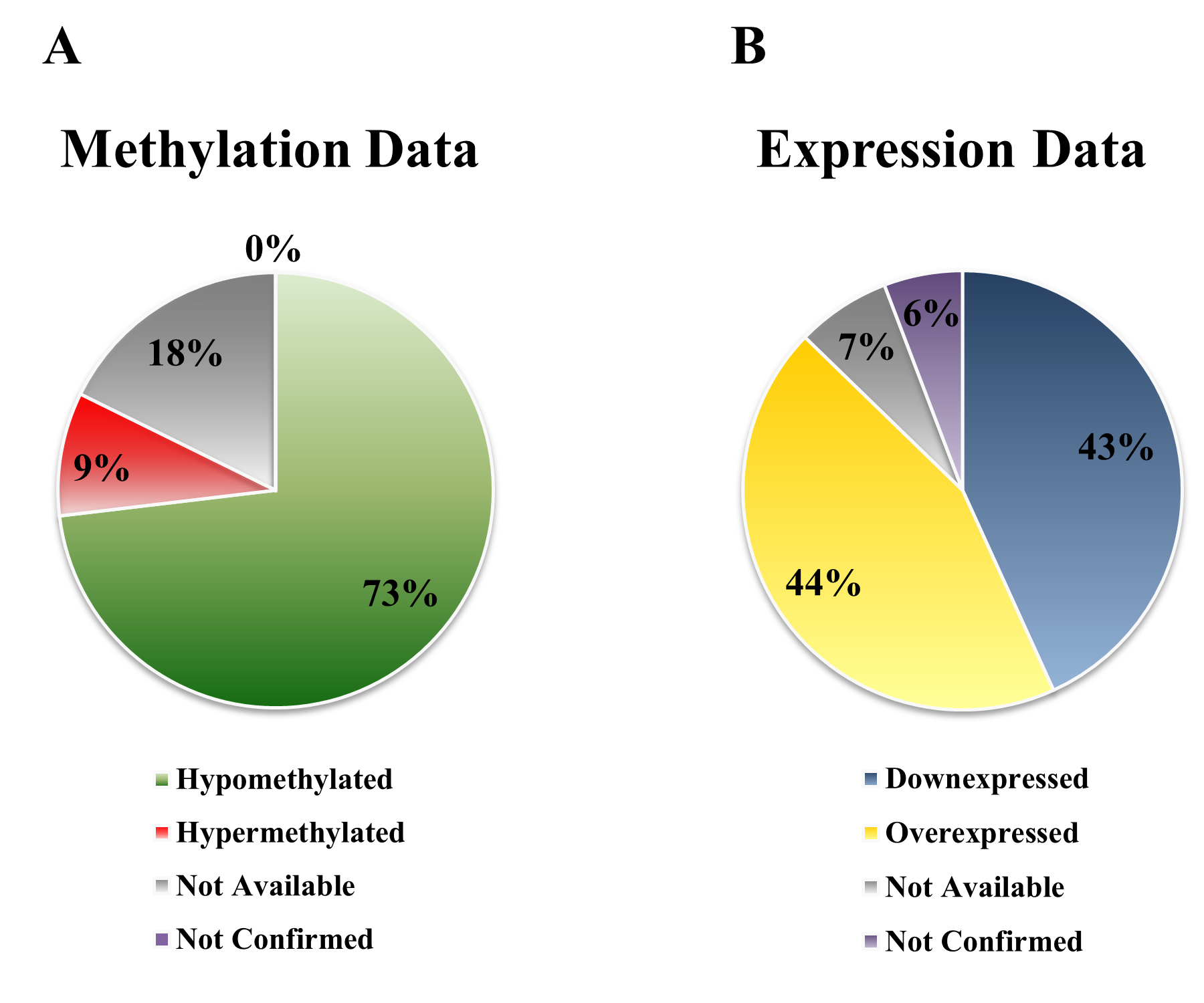
**

**Supplementary Figure 2.** FGF canonical signaling pathway potentially activated in PTC. Dark green molecules indicate genes downexpressed and hypermethylated in PTC samples. Dark red molecules indicate up-regulated and hypomethylated genes. Light red molecules represent overexpression and light green molecules represent downexpression. Both colors in the same molecules indicate different members of the same family with contrary expression levels.


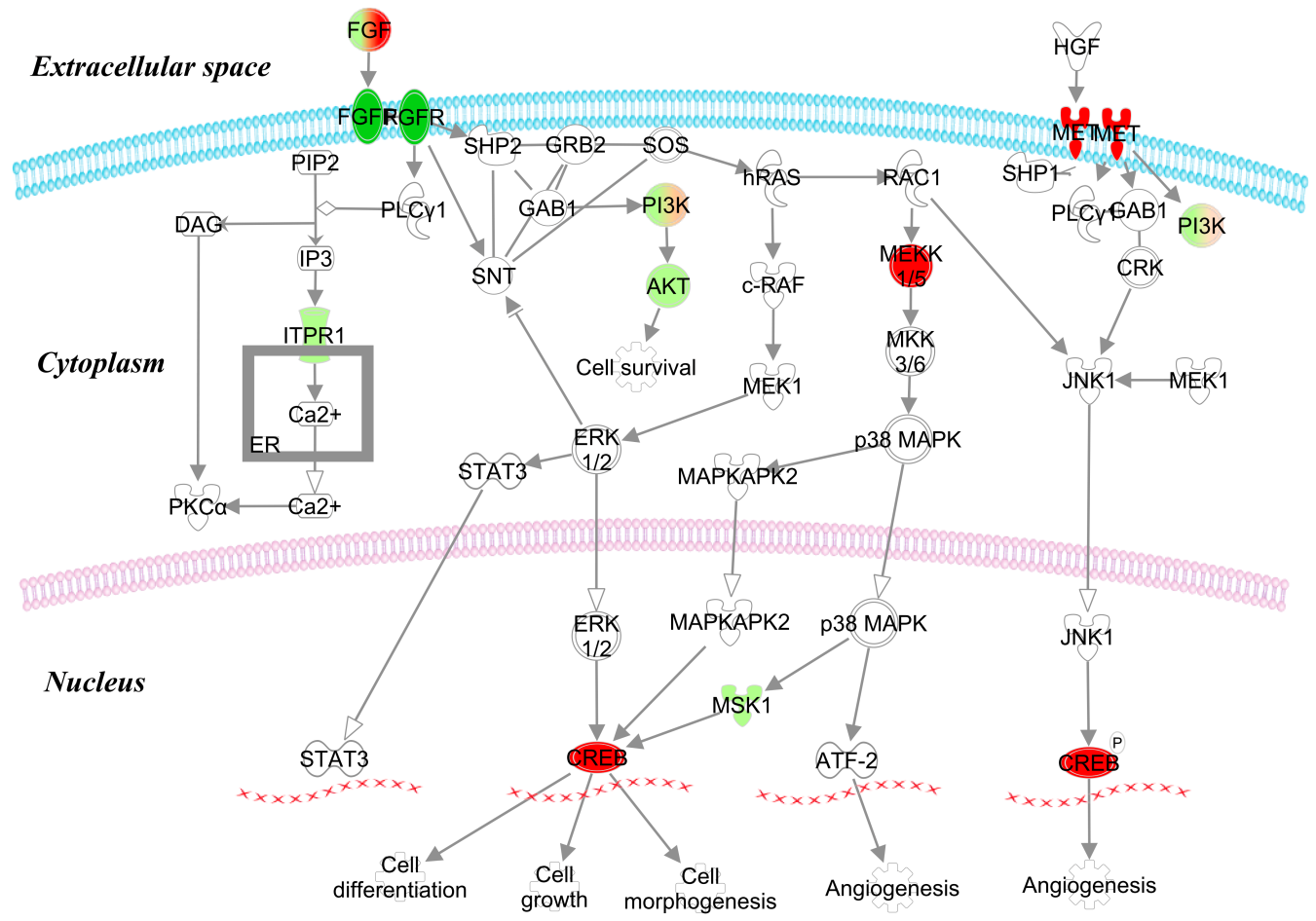


**Supplementary Figure 3.** (**A**) DNA methylation and (**B**) gene expression levels detected in the selected genes according to *BRAF*V600E mutation. **A.** *FGF1*, *GABRB2* and *RDH5* hypomethylation were associated with *BRAF*V600E PTC samples by pyrosequencing. **B.** *ERBB3* and *GABRB2* overexpression were associated to *BRAF*V600E PTC samples by RT-qPCR.

**
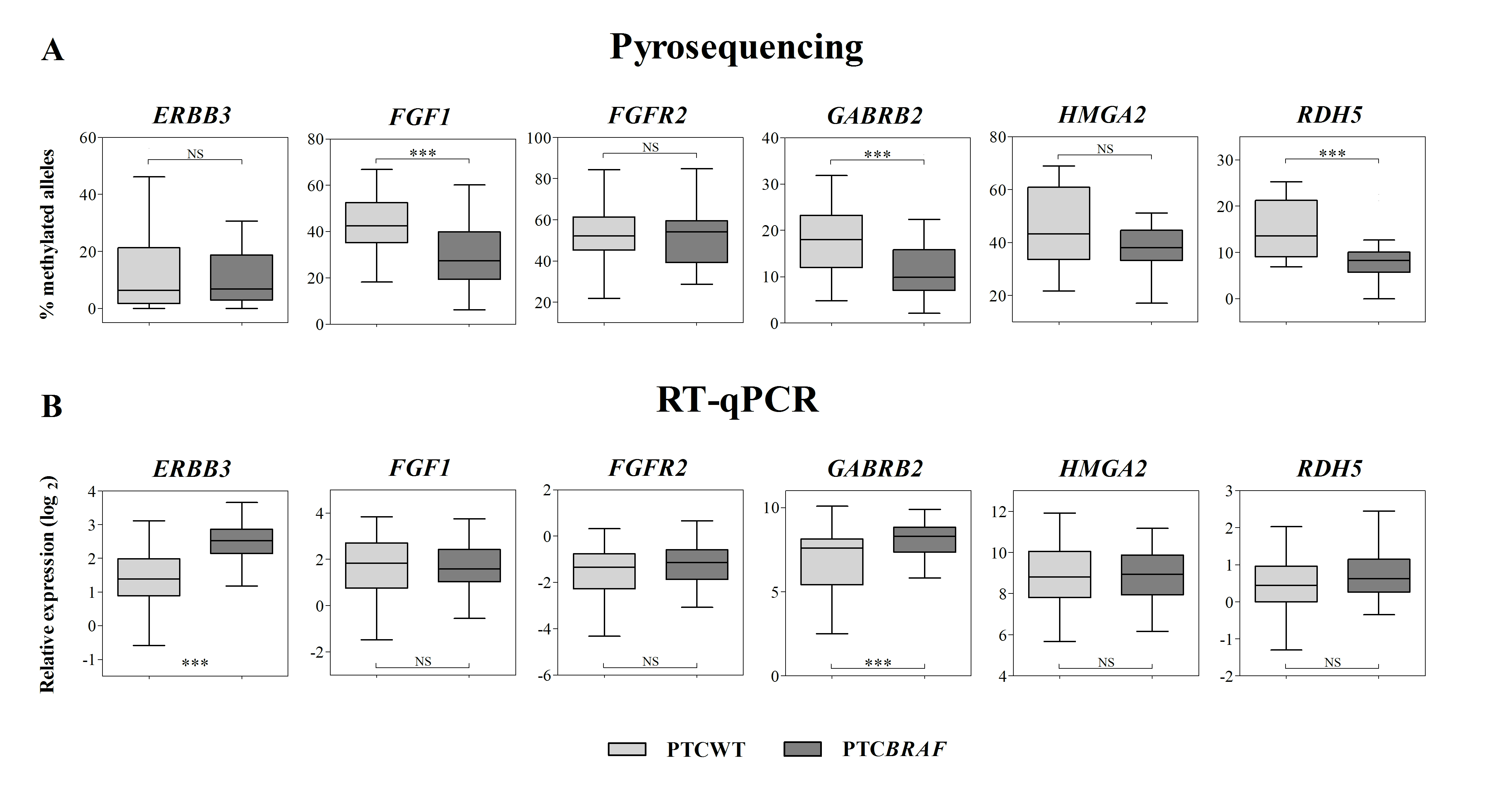
**
